# The Role of FODMAPs in Sports Nutrition: A Narrative Review and Clinical Implications

**DOI:** 10.3390/nu18020239

**Published:** 2026-01-12

**Authors:** Aleksandra Kołodziejczyk, Wiktoria Staśkiewicz-Bartecka, Marek Kardas

**Affiliations:** Department of Food Technology and Quality Evaluation, Department of Dietetics, Faculty of Public Health in Bytom, Medical University of Silesia in Katowice, ul. Jordana 19, 41-808 Zabrze, Poland; mkardas@sum.edu.pl

**Keywords:** FODMAP, endurance athletes, exercise-induced gastrointestinal syndrome, sports dietetics, carbohydrate malabsorption, gastrointestinal distress

## Abstract

Background/Objectives: Intense physical activity can cause gastrointestinal symptoms, negatively impacting athletic performance. A low-FODMAP diet has the potential to reduce these symptoms and is increasingly being considered by physically active individuals. The aim of this review is to present the current knowledge on the importance of FODMAPs in sports nutrition. Methods: A narrative review was conducted in PubMed, Web of Science, ScienceDirect, and Google Scholar, covering publications published up to October 2025. Original studies, reviews, and meta-analyses addressing the relationship between FODMAP intake and gastrointestinal symptoms during physical activity were included. Selected articles were assessed for specific criteria, and the results were grouped thematically to present the current state of knowledge. Results: FODMAP consumption increases the risk of intestinal symptoms. Short-term FODMAP restriction, especially before and during exercise, reduced the severity of symptoms in most of the analyzed studies. Data on the long-term effects of a low FODMAP diet on the health, nutrition, and gut microbiota of athletes remain limited. Conclusions: A strategy of short-term FODMAP restriction in athletes’ diets shows potential for reducing gastrointestinal symptoms. An optimal approach requires individualization. Further research is needed to monitor potential side effects and long-term outcomes.

## 1. Introduction

Low physical activity, due to its significant impact on the development of chronic diseases, is currently a major public health challenge [1,2,3]. With increasing awareness of the importance of regular physical activity for health, there is a growing interest in recreational sports [1]. Although physical activity offers numerous health benefits, intense exercise can be associated with gastrointestinal symptoms, such as abdominal pain, bloating, diarrhea, and discomfort [4,5,6]. These symptoms affect not only the comfort and effectiveness of training but also the overall health and quality of life of physically active individuals. Appropriate nutritional strategies play a crucial role in preventing these disorders [4,5,7]. Carbohydrates are the primary source of energy during physical exercise, and their adequate intake is essential [8]. For activities lasting longer than 60 min, carbohydrate supplementation can delay the onset of fatigue and improve exercise tolerance [8,9]. It is worth noting, however, that many popular carbohydrate snacks, such as gels, bars, and energy drinks [8,10], may contain FODMAPs (Fermentable Oligosaccharides, Disaccharides, Monosaccharides, and Polyols) [11].

FODMAPs are short-chain carbohydrates (CHO) characterized by poor absorption in the small intestine and high susceptibility to bacterial fermentation in the large intestine [7,12]. They include fructans (FOS), galactooligosaccharides (GOS), lactose, excess fructose, and polyols [13,14]. Their consumption, despite their potential benefits [12,15], may contribute to the exacerbation of gastrointestinal symptoms [13,14,16]. These symptoms result from both increased fermentation and an osmotic effect, leading to increased water influx into the intestinal lumen and the overproduction of short-chain fatty acids [7,12]. During intense exercise, when gastrointestinal tolerance is reduced [17], these processes can significantly reduce gastrointestinal comfort and exercise tolerance.

Individual FODMAP fractions exhibit diverse physiological properties. Oligosaccharides, including FOS and GOS [16,18,19], present in rye, garlic, pistachios, watermelon, artichoke, barley, wheat, leek, onion, asparagus, cabbage, brussels sprouts, some nuts, seeds, legumes, dairy products, and chicory [16,20,21,22,23], have prebiotic effects [16], but their fermentation can induce flatulence and abdominal discomfort even in healthy individuals [13,14]. Among the disaccharides, lactose is of particular importance [14,24,25], the sources of which include yogurt, buttermilk, milk, and cottage cheese [26]. Lactose consumption, especially in people with lactase deficiency, increases the flow of water into the intestine and undergoes bacterial fermentation, intensifying gastrointestinal symptoms [14]. In the context of sports nutrition, the use of fructose and glucose mixtures is recommended [9,27], however, excess fructose consumed in relation to glucose has a slower absorption rate [12,20,28], which favors the osmotic effect [14]. Polyols, in particular sorbitol and mannitol [13,16,29], present in some fruits [30,31], vegetables [31], mushrooms, marine algae, tree secretions [32] and used as laxatives [30] and sweeteners with a zero glycemic index [33], due to poor absorption [30], intensify the osmotic effect and fermentation in the large intestine, which may also influence the occurrence of symptoms [14,20].

Consuming foods rich in FODMAPs exacerbates gastrointestinal symptoms, which is important not only for physical performance but also for gastrointestinal health and quality of life [34,35]. Currently, the role of a low FODMAP diet is increasingly emphasized, as it has a beneficial effect on reducing gastrointestinal symptoms in selected conditions, and its use is also being considered among physically active individuals [4,7,36,37].

The literature contains reviews of gastrointestinal symptoms in athletes and the impact of selected nutritional strategies, including the low-FODMAP diet. However, there is still a lack of studies that synthesize these issues in the context of sports nutrition practice, integrating clinical, practical, and regulatory perspectives, with a particular focus on short-term interventions, exercise nutrition, and implementation constraints. Therefore, this review aims to organize the current data on the importance of FODMAPs in sports nutrition, with particular emphasis on their impact on gastrointestinal function and the potential clinical significance of a low FODMAP diet in the prevention and alleviation of gastrointestinal disorders in physically active individuals.

## 2. Materials and Methods

The growing interest in assessing the effectiveness of the low FODMAP diet among endurance athletes was the primary motivation for preparing this review. Although this diet has been widely studied in the general population, available data regarding its effectiveness in the athletic setting are still limited. To address this gap, this paper is a narrative review and focuses on synthesizing the current evidence regarding the importance of fermentable oligosaccharides, disaccharides, monosaccharides, and polyols in modulating gastrointestinal symptoms in physically active individuals. Due to the diverse nature of studies on athlete populations, dietary interventions, and methods of assessing gastrointestinal symptoms, a narrative approach was adopted to enable synthetic integration of diverse data and their practical interpretation while avoiding the limitations typical of systematic reviews that require a high degree of data homogeneity.

### 2.1. Review Procedure

The review began with a preliminary analysis of the literature related to the topic and aim of the study. This allowed for the identification of key research areas and the selection of keywords relevant to the study’s topic. A systematic search of publications was then conducted in the PubMed, Web of Science, ScienceDirect, and Google Scholar databases, covering articles published up to October 2025, without geographical restrictions.

The search was based on predefined keyword combinations, selected based on their relevance to the topic of fermented oligosaccharide, disaccharide, monosaccharide, and polyol consumption in the context of physical activity and gastrointestinal symptoms in endurance athletes. The following terms were used in various combinations during the search: (“FODMAP” OR “fermentable oligosaccharides, disaccharides, monosaccharides, and polyols” OR “low FODMAP diet” OR “low fermentable oligosaccharides, disaccharides, monosaccharides, and polyols diet” OR “high FODMAP”) AND (“exercise” OR “athletes” OR “endurance athletes” OR “runners” OR “cyclists” OR “triathletes” OR “physical activity” OR “sports”) AND (“gastrointestinal symptoms” OR “gastrological disorders” OR “digestive health” OR “GI distress” OR “GI complaints” OR “digestive discomfort” OR “exercise-induced gastrointestinal”).

The reference lists of the selected articles were also analyzed during the selection process to identify publications potentially missed in the initial search. All search terms were internally reviewed by the authors for terminological consistency and thematic fit with the scope of the review.

### 2.2. Eligibility Criteria and Search Strategy

Publications meeting the following criteria were eligible for the review: (1) they were published between January 2005 and October 2025, (2) were available in full text in English, (3) were classified as original research, review articles, or meta-analyses, and (4) addressed issues related to FODMAPs and/or gastrointestinal disorders in the context of physical activity.

Publications that did not meet the scientific or thematic requirements were excluded from the analysis, including: (1) non-peer-reviewed articles, (2) commentaries, letters to the editor, and conference reports, (3) studies conducted exclusively on animal or in vitro models, and (4) publications that did not address the relationship between diet, physical activity, and gastrointestinal function.

The selection process was completed in November 2025. Each publication was assessed for methodological reliability, transparency of reporting, and clinical relevance. Due to the diverse nature of the studies analyzed, a narrative synthesis of the results was used. The source data were grouped into key thematic areas, including:

(1) Epidemiology and characteristics of gastrointestinal disorders in athletes,
(2) Pathophysiology of exercise-induced symptoms and the role of FODMAPs in their modulation,
(3) Nutrition strategies in sports that incorporate FODMAP restriction,
(4) Clinical implications and practical importance of dietary interventions in the care of athletes.

This thematic categorization facilitated the integration of research findings from various disciplines and research projects into a coherent, comprehensive framework.

The database search initially identified 412 records across PubMed, Web of Science, ScienceDirect, and Google Scholar. After the removal of duplicates, 356 records remained for title and abstract screening. At this stage, 243 articles were excluded due to a lack of relevance to physical activity or sports nutrition, non-human study design, or publication in non-peer-reviewed sources.

The full texts of 113 potentially eligible articles were subsequently assessed for compliance with the predefined inclusion criteria. Following full-text evaluation, 40 articles were excluded, primarily due to insufficient focus on FODMAP-related outcomes, inadequate methodological transparency, or lack of relevance to athletic populations. Ultimately, 73 publications met all inclusion criteria and were included in the final narrative synthesis.

### 2.3. Quality Assessment and Selection of Publications

All publications were subjected to a qualitative assessment without the use of formal scoring tools. A key eligibility criterion was publication in peer-reviewed scientific journals characterized by a high level of methodological transparency. The selection process was conducted independently by two authors (A.K. and W.S.-B.) and performed in two stages. In the first stage, titles and abstracts were screened independently to identify potentially relevant studies and to exclude publications that were not aligned with the subject area, did not meet the predefined inclusion criteria, or were published in non-peer-reviewed sources. In the second stage, full-text articles were assessed independently for methodological quality, transparency of reporting, and relevance to the scope of the review.

Any discrepancies between reviewers at either stage were resolved through discussion. If consensus could not be reached, a third author (M.K.) was consulted to make the final decision. Only studies meeting all predefined inclusion criteria and demonstrating high methodological quality were included in the final analysis. As a result of this selection process, 73 publications were considered the most current, reliable, and relevant to the clinical and nutritional implications of a low FODMAP diet in athletes. This review excluded non-English language publications and gray literature, which may partially limit the comprehensiveness of the findings. Furthermore, due to the narrative nature of the study, a formal meta-analysis was not conducted, which may affect the generalizability of the results.

Due to the narrative nature of this review, no formal methodological quality assessment tools or reporting guidelines were applied. Instead, methodological quality was assessed qualitatively based on predefined criteria, including clarity of study objectives, appropriateness of study design, transparency of methodology, adequacy of data reporting, and relevance to the research question. Clinical relevance was defined as direct applicability of study outcomes to physically active individuals or athletes, including implications for exercise-related gastrointestinal symptoms, dietary strategies, or practical nutritional recommendations in sports settings. Formal risk-of-bias assessment was not performed due to the narrative nature of the review and the heterogeneity of included study designs; instead, potential methodological limitations were considered qualitatively during data interpretation.

## 3. Gastrointestinal Problems Among Endurance Athletes

Gastrointestinal (GI) symptoms experienced by competitive athletes are a common phenomenon today [11,34,36]. These symptoms significantly impair physical activity and negatively impact performance [7,37]. However, the gastrointestinal consequences can extend far beyond simply impairing athletic performance. They can impact an athlete’s mental well-being to the point of discontinuing their sport [17]. It should be emphasized that these symptoms vary depending on the severity of the symptoms. They can range from minor and minimally impacting the athlete to severe and significantly impacting the athlete’s performance [7,11]. It is currently believed that gastrointestinal symptoms stem from the simultaneous occurrence of physiological, mechanical, psychological, and nutritional problems [11].

### 3.1. Frequency of Symptoms

Gastrointestinal symptoms are among the most frequently reported phenomena associated with endurance sports. Studies conducted over the past few years have revealed that such disorders are observed in a significant percentage of athletes, regardless of whether they compete in team or individual sports (Table 1). Furthermore, this problem affects athletes who train regularly, regardless of their background, type of activity, or level of advancement.

The data indicate that gastrointestinal symptoms affect between half and two-thirds of active athletes. These studies emphasize that these disorders occur both immediately after and during training [38,39,40,41,42,43,44,45,46,47,48,49]. It is also significant that the obtained results are similar regardless of the country where the studies were conducted. This indicates the universality of pathophysiological factors directly related to the body’s response to exercise and high intensity, which contribute to these disorders.

The results of similar studies conducted in China differ from the above results. The percentage of athletes who experienced gastrointestinal problems was reported to be as high as 26% [38]. This difference may be due to the way symptoms were reported, the reliability of the responses, or different dietary habits in that region. However, this is the only instance in which international studies reported a different incidence of the analyzed disorders. Differences in the frequency of symptoms between individual disciplines and populations are presented in Table 1.

Among the subgroups most severely affected by GI symptoms, runners with irritable bowel syndrome (IBS) should be highlighted. In this group, gastrointestinal symptoms affect up to 94% of athletes, while only 54% of runners without IBS experience similar problems [44]. This correlates with the results obtained in triathletes and ultramarathoners, where gastrointestinal symptoms among athletes without IBS were reported by slightly over 60% of participants [39,42]. Similar results were also observed in studies among team sports athletes and cyclists [40,47]. This only supports the claim that this phenomenon is independent of the specific discipline and universal.

Differences between studies include not only the definitions of gastrointestinal symptoms and their recording methods, but also the type of sport practiced and the duration and intensity of exercise. These factors can constitute a significant source of variability in the frequency of reported GI complaints. For example, long-distance running may be associated with a different symptom profile than cycling, and shorter interventions may not reveal all the potential effects of training or diet. Consequently, direct comparison of results between studies requires consideration of these methodological differences. Despite these discrepancies, the available data are consistent, and their analysis suggests that gastrointestinal symptoms are a significant issue related to endurance sports. This issue should be considered when planning training and related nutritional aspects.

### 3.2. Characteristics of Symptoms

Although balanced physical activity has a protective effect on the gastrointestinal tract, endurance training has a negative impact. The decrease in blood flow to the intestines associated with prolonged exercise leads to the generation of ischemic damage, increasing intestinal permeability and mucosal integrity. This leads to the phenomenon of “leaky gut” [50]. The symptoms associated with physical activity above-mentioned can significantly impact athletic performance [4].

Acute upper gastrointestinal symptoms are primarily characterized by the occurrence of reflux, vomiting, nausea, heartburn, belching, and chest pain [4,5,11]. They are reported by a significant number of endurance athletes. In observational studies, the percentage of individuals experiencing these symptoms varies depending on various factors, with nausea and gastric regurgitation being among the most frequently reported symptoms [39,41,45].

When analyzing lower gastrointestinal symptoms, the most important ones include bloating, rectal bleeding, diarrhea, abdominal cramps, and the need to defecate [4,5,11]. These symptoms are more serious than those originating in the upper gastrointestinal tract. They can primarily impair an athlete’s performance [17]. Lower gastrointestinal symptoms in athletes are relatively common and include both mild symptoms and more severe symptoms that may require exercise interruption [38,39,40,41,42,43,44,45,46,47,48,49], with bloating being one of the most frequently reported symptoms [38,40,44,45]. Prompt diagnosis and treatment can help reduce gastrointestinal disorders and inflammation associated with chronic diseases [50].

### 3.3. Mechanisms of Digestive Discomfort During Exercise

Gastrointestinal disorders occurring during endurance activity are associated with a complex combination of diverse factors. They affect both the lower and upper gastrointestinal tract [7]. Pathophysiology involves multifactorial interactions between the gastrointestinal tract and the intestinal, circulatory, and immune systems, as well as the central nervous system [17]. In the conditions of training and sports competition, mechanical intestinal damage, ischemia, and the secretion of neuroendocrine substances are of particular importance [7,50]. Exercise-related stress is also important. Its occurrence results from adaptations in two physiological pathways, which in turn influence the function and integrity of the gastrointestinal tract [4].

During intense exercise, blood flow is redistributed from the gastrointestinal tract toward tissues with increased activity during physical activity (e.g., the thermoregulatory system or working muscles), which results in a significant decrease in splanchnic blood flow, reaching up to 80% of resting values [4,7,17]. The consequences of this phenomenon are ischemic damage, increased intestinal permeability, and activation of the inflammatory response [6,17,51]. In practice, these phenomena promote bacterial translocation and may negatively affect both exercise capacity and regenerative processes by impairing the absorption of fluids, electrolytes, and nutrients and by increasing pain [4,17,51].

Physical exercise also activates the sympathetic nervous system and releases stress hormones, which in athletes translates into disturbances in gastrointestinal motility and intestinal transit time [4,17]. The exact intensity and duration of exercise have varying effects on the enteric nervous system. Exercise below 60 min appears to be beneficial for gastrointestinal motility, while exercise above 90 min inhibits this phenomenon [17]. The situation is similar with exercise intensity. Low-intensity exercise positively affects motility, while high-intensity exercise inhibits it [17,51,52]. It should also be noted that endurance activities also inhibit gastric motility through the associated sympathetic activation and stress [17].

An increase in the duration and intensity results in a corresponding increase and exacerbation of symptoms [4,50]. In the long-term, chronic gastrointestinal symptoms can lead to negative health consequences. Furthermore, this makes them a factor limiting physical performance [53]. The risk of symptoms during physical activity is influenced by many modifiable factors [54], including inappropriate nutritional strategies (e.g., consuming significant amounts of fat and fiber immediately before exercise), inadequate hydration, which can exacerbate intestinal cramps and diarrhea [50], as well as sleep deprivation and increased stress [6,54]. Gastrointestinal symptoms then resemble irritable bowel syndrome (IBS) and other functional gastrointestinal disorders [17]. It should therefore be emphasized that the proper management of diet, hydration, training time and intensity, and mental stress are important not only for improving athletic performance but also for maintaining the health of the digestive system in athletes.

## 4. The Importance of FODMAPs in Sports Nutrition

Nutritional factors are a key factor in the gastrointestinal tolerance of prolonged exercise. A properly composed diet significantly contributes to reducing the reactions associated with training load [51]. Particular attention in this regard should be paid to fermentable carbohydrates [17]. Their presence is common in products related to peri-exercise nutrition for endurance athletes [11]. The specific properties of fermentable carbohydrates mean that these compounds have the potential to modify intestinal contents. Additionally, they influence gas production and gastrointestinal motor function [14,16]. They increase the possibility of nutrient penetration into the ileum, which further negatively impacts the severity of symptoms during athletic activity [34]. Intense physical activity, with subsequent impairment of the function and integrity of the gastrointestinal tract (i.e., transporters), leads to a situation in which undigested food particles, such as FODMAPs, can increase fecal volume and translocate osmotic water, leading to physiological consequences, including diarrhea or loose stools [36,38].

It should be emphasized that during intense physical activity, the described mechanisms are intensified, which increases the risk of ailments [55]. Assessing the role of FODMAPs in the diet of endurance athletes is therefore a key aspect that must be considered in developing effective nutritional responses.

### 4.1. FODMAP Consumption in Athletes’ Diets

Diet, as the foundation of an effective training program, allows athletes to achieve the desired gains. Its most important element is the supply of carbohydrates, which are a source of energy [56]. The role of this nutrient as a key element of an effective training diet has been sufficiently documented [9,57]. The source of this crucial role of carbohydrates lies in their ability to facilitate more effective regeneration and optimize glycogen stores while being readily available [58]. Glycogen plays a significant role during physical activity. It should be emphasized that the higher the glycogen content in an athlete’s muscles, the greater their exercise performance [59]. Currently, carbohydrate (CHO) supply is a key element of peri-exercise nutrition. The amount of carbohydrate intake depends on the intensity and duration of the activity [8].

According to currently available nutritional guidelines, carbohydrate consumption is recommended during exercise, depending on its duration and intensity [9]. Reduced fatigue in athletes supplementing with carbohydrates during exercise is associated with reduced liver glycogen storage, increased oxidation rates, and significantly delayed muscle glycogen depletion [60]. Notably, many sports nutrition products contain substantial amounts of FODMAPs. This applies to both products used during exercise and those recommended for pre-competition consumption. This is particularly harmful to sensitive digestive systems [11]. Research by Killian et al. [11] revealed that as many as 60% of athletes consumed high-FODMAP products during their pre-competition diet. Of the sixteen products intended for athletes, seven were found to contain high FODMAPs in a single serving. Even the remaining products contained FODMAPs in a single serving at a concentration that, if consumed in multiple servings (as occurs during competition), would result in excessive FODMAP intake [11].

### 4.2. The Low FODMAP Diet in the Context of Physical Activity

Reducing FODMAP intake during endurance sports activity is a potential strategy for reducing the severity of gastrointestinal symptoms and even minimizing their frequency. This is associated with slowing gastrointestinal motility, which favorably reduces the ability of the malabsorption of nutrients to reach the ileum [34,37]. Therefore, it should be noted that a low FODMAP diet appears to be an effective strategy for reducing gastrointestinal symptoms [15].

The dietary strategy of reducing FODMAP intake is reflected in the development of a low FODMAP diet. It is important to note that this also affects the gut microbiota. According to the results of the study by Gaskel et al. [61], short-term intervention changes the relative abundance of certain bacteria in the stool—the Firmicutes population increases and the Bacteroidota population decreases. These changes may affect fermentation and the production of short-chain fatty acids (SCFAs) [61]. It should be emphasized, however, that short-term interventions using the above diet do not increase the risk of bacterial translocation into the bloodstream during activity, which may suggest the safety of such an intervention [5].

The low FODMAP diet was originally developed to reduce the symptoms of irritable bowel syndrome [62]. Its foundation is the maximum possible elimination of fermentable short-chain carbohydrates [63]. According to research, the low FODMAP diet positively affects the athletic performance of endurance athletes, primarily by reducing symptoms [36,64]. It is particularly recommended for athletes with non-specific digestive symptoms, including irritable bowel syndrome [36]. However, despite the health benefits, current use of the low FODMAP diet among athletes is relatively low. In a study of 137 athletes training in triathlon, running, and other disciplines lasting more than 60 min, only 5.4% of participants followed the low FODMAP diet [65]. This may indicate low public awareness of the possible benefits of using this strategy in minimizing gastrointestinal symptoms.

#### 4.2.1. Strategies for Implementing a Low FODMAP Diet for Athletes

The low FODMAP diet is based on eliminating fermentable oligosaccharides, di-, monosaccharides, and polyols over a four- to eight-week cycle, and then reintroducing them into the diet, taking into account individual tolerance [12].

Regarding the low FODMAP strategy in athletes, it should be emphasized that, according to current research, reducing FODMAP intake 24–48 h before physical activity significantly helps to minimize gastrointestinal symptoms [66,67]. However, implementing a low FODMAP diet in athletes requires appropriate modifications to the base low FODMAP diet due to the potential occurrence of gastrointestinal symptoms during activity. Measures are necessary to reduce this risk while maintaining an adequate carbohydrate intake [68]. Furthermore, interventions planned several days or weeks in advance allow for more precise adjustment of FODMAP intake to the individual athlete’s tolerance [69,70]. This allows for greater control over the occurrence of symptoms during competition.

The results of studies on the effects of a low FODMAP diet on athletes with varying durations of activity are presented in Table 2. Due to the diversity of research designs, the obtained results require interpretation taking into account potential methodological variability. Despite these limitations, analysis of the compiled data indicates a consistent trend that short-term implementation of a low-FODMAP diet leads to a reduction in the severity of gastrointestinal symptoms in endurance athletes, regardless of the intensity of physical activity.

#### 4.2.2. Long-Term Efficiency and Safety

The long-term safety of the low FODMAP diet for athletes remains insufficiently researched, despite the recent increase in interest in this method [12,62,63]. However, at this stage of the discussion, it is necessary to point out that due to the restrictive nature of the low FODMAP diet, its long-term use, if improperly balanced, may contribute to problems related to insufficient energy and carbohydrate intake as well as promote eating disorders. Carbohydrate deficiency may negatively impact performance, recovery, and the body’s adaptation to endurance training. Furthermore, limited energy intake may undermine the potential health benefits of reduced gastrointestinal symptoms [10,11,63]. The restrictive phase is not intended to be used on a long-term basis [12], and its prolongation may pose a risk of energy and macronutrient deficiencies, which are a key source of energy for athletes.

As suggested in the literature, permanent reduction or even excessive prolongation of the reduction in fermentable oligo-, di-, and monosaccharides, and polyols in the diet raises significant doubts in the context of safety related to the diversity of the intestinal microbiome [12,62,63,71]. Reducing the supply of FODMAPs leads to a reduction in the substrates necessary for the development and activity of beneficial probiotic bacteria, including Bifidobacterium and Lactobacillus [12]. As indicated in the article by Bellini et al., analyses related to the intestinal microbiota and its metabolic products yield mixed and sometimes contradictory results [12]. It is worth noting, however, that most existing studies are based on clinical populations, primarily individuals with IBS, rather than on healthy athletes [12]. This limits the ability to directly generalize the results to the sports environment, where specific physical and metabolic demands may influence the microbiota response [72]. Therefore, the interpretation of existing data requires caution. Observed microbiota changes and potential health consequences in clinical populations may not fully reflect the situation in the sports environment.

Therefore, a selective strategy seems to be the most optimal. Following a low FODMAP diet only during the pre-race period avoids the risk of the effects of reduced dietary carbohydrate and energy intake [11]. This correlates with research findings that a time-limited low FODMAP diet has a positive impact on reducing digestive symptoms during endurance physical activity [66,67,69,70]. Interventions conducted among endurance athletes to date are short-term and are focused on reducing symptoms, not on long-term effects.

Due to the small sample sizes, it is important to emphasize the need for further research, not only on short-term interventions but also on the long-term effects of the low FODMAP diet on the microbiota. This is essential to fully determine its safety for athletes’ health.

#### 4.2.3. Practical Constraints and Challenges

Adopting a low FODMAP diet in the diets of athletes in various sports disciplines faces numerous limitations. This is related to both legal issues and practical application.

First, the lack of a legal definition of the term FODMAP in European Union law is worth noting. This results in the lack of a formally required declaration of FODMAP content by the manufacturer of a given product. This creates a market situation in which access to certified low FODMAP products is extremely limited [13]. Those interested in implementing this type of strategy are forced to rely on incomplete data from manufacturers [11] or on data published by research centers specializing in FODMAP analysis. This is a significant impediment, particularly in the context of products such as isotonic drinks, recovery drinks, gels, and bars, which are intended for use during exercise and often contain concentrated sources of carbohydrates with varying degrees of fermentation. The introduction of uniform regulations and labelling of FODMAP content could significantly facilitate diet planning for athletes and specialists in sports nutrition, increasing the precision of product selection, improving nutritional safety, and enabling more effective management of gastrointestinal symptoms during training and competitions.

Another challenge for athletes wishing to implement a low FODMAP strategy is ensuring an adequate supply of energy and carbohydrates, despite the need to reduce FODMAP-rich products, which dominate the market in this category. A properly balanced athlete’s diet is characterized by a high carbohydrate requirement [56]. Implementing low-FODMAP recommendations may result in an undesirable decrease in the diet’s caloric value. It may also make it difficult to ensure the proper supply of easily digestible carbohydrate sources [7]. This phenomenon is particularly intensified during periods of high training load. Maintaining adequate glycogen stores is then essential to ensure proper recovery and high performance [73].

Difficulties in implementing a low FODMAP diet also stem from the need for proper meal planning. Advanced nutritional knowledge is required, including individual tolerance to specific FODMAP groups. Implementing a system for monitoring gastrointestinal symptoms becomes essential [63]. Implementing this type of activity is time-consuming, while endurance training itself significantly limits an athlete’s available time. Travel, competition days, and schedule variability also pose challenges, complicating the entire situation logistically.

## 5. Conclusions

Gastrointestinal complaints among endurance athletes remain a significant issue, impacting their comfort, training effectiveness, and performance. Implementing a low FODMAP strategy has the potential to reduce gastrointestinal symptoms in athletes, particularly when implemented shortly before and during exercise. However, despite the significant potential and promising current results, further research and knowledge are needed, particularly regarding the long-term impact of a low FODMAP strategy on health and nutrition, as well as the gut microbiota [12,62,63].

Implementing a low FODMAP strategy may be considered both in athletes diagnosed with irritable bowel syndrome (IBS) and in athletes without IBS who experience gastrointestinal symptoms that affect comfort during physical exercise. The most sustainable approach currently appears to be implementing a low FODMAP diet for short, strictly controlled, and targeted periods while tailoring recommendations to individual needs. A sustained assessment of potential side effects is also essential.

In practice, this indicates the need for close supervision of the implementation of this strategy by qualified specialists, particularly sports dietitians, to ensure the safety of the intervention, reduce the risk of energy and carbohydrate deficiencies, and minimize potential adverse changes in the gut microbiota. Individualizing nutritional management, taking into account the specific discipline, the athlete’s preferences, and their susceptibility to gastrointestinal complaints, is a key element in optimizing training outcomes and exercise comfort.

## Figures and Tables

**Table 1:** The frequency of gastrointestinal symptoms in athletes of various disciplines.

Study Location	Sport Discipline	Sample Size (n)	Prevalence of GI Symptoms (%)	References
China	Running	805	26.1	Zhao et al., 2025 [38]
Spain	Triathlon, Running	42	61.9	Jimenex-Alfageme et al., 2024 [39]
Spain	Cycling	138	48.6	Martinez et al., 2025 [40]
Netherlands	Running	433	40	ten Haaf et al., 2014 [41]
Netherlands	Running	252	89.3	Hoogervorst et al., 2019 [42]
Netherlands	Running	1281	45.2	Ter Steege et al., 2008 [43]
Netherlands and Belgium	Running	306 (153 IBS +153 without IBS)	94 (IBS); 54 (without IBS)	Baart et al., 2024 [44]
Netherlands and Belgium	Running	1993	57	Baart et al., 2023 [45]
Ireland	Ultramarathon	68	61.8	Ryan et al., 2024 [46]
Canada	Individual and team sports	96	66	Jamieson et al., 2025 [47]
USA (Phoenix, Arizona)	American football	44	52	Wardenaar et al., 2023 [48]
Great Britain and Ireland	Endurance sports (elite athletes)	400	53.3	Kearns et al., 2025 [49]

**Table 2:** Effects of low FODMAP dietary interventions in physically active individuals.

Exercise Type	Sample Size (n)	Intervention	Intervention Duration	Main Results	References
Continuous running for 2 h under heat stress conditions (35.6 °C)	13	Low FODMAP diet compared to high FODMAP diet	24 h	No significant differences in plasma microbiome after exercise; significant differences in the relative number of bacteria and the concentration of short-chain fatty acids in feces	Gaskell et al., 2023 [61]
Continuous running for 2 h under heat stress conditions (35.6 °C)	18	Low FODMAP diet compared to high FODMAP diet before 2 h of running at 35 °C	24 h	Stronger gastrointestinal symptoms and increased intestinal fermentation after a high FODMAP diet	Gaskell et al., 2020 [67]
Continuous 2 h running	12	High carbohydrate diet: low FODMAP compared to high FODMAP	48 h	Lower severity of gastrointestinal symptoms before and during exercise following the low FODMAP diet	Scrivin et al., 2024 [66]
56 km ultramarathon	44	Assessment of FODMAP intake and carbohydrate malabsorption	3 days	No association between FODMAP intake or carbohydrate malabsorption and gastrointestinal symptoms	Convit et al., 2024 [34]
Running training at varying intensities	11	Low FODMAP diet compared to high FODMAP diet	6 days	Reduced bloating, diarrhea, loose stools, and urge to defecate after the low FODMAP diet	Lis et al., 2018 [70]
Running training	16	Low FODMAP diet compared to high FODMAP diet	7 days	Significant reduction in gastrointestinal symptoms following the low FODMAP diet	Wiffin et al., 2019 [69]

## Data Availability

No new data were created or analyzed in this study. Data sharing is not applicable to this article.

## Abbreviations

The following abbreviations are used in this manuscript:

FODMAP	Fermentable Oligosaccharides, Disaccharides, Monosaccharides and Polyols
GI	Gastrointestinal
IBS	Irritable Bowel Syndrome
GM	Gut Microbiota
GOS	Galactooligosaccharides
g	Gram
FOS	Fructooligosaccharides
GLUT-2	Glucose Transporter Type 2
GLUT-5	Glucose Transporter Type 5
CHO	Carbohydrates
SCFAs	Short-Chain Fatty Acids
h	Hour
km	Kilometer
